# Encrypting messages with artificial bacterial receptors

**DOI:** 10.3762/bjoc.16.225

**Published:** 2020-11-12

**Authors:** Pragati Kishore Prasad, Naama Lahav-Mankovski, Leila Motiei, David Margulies

**Affiliations:** 1 Department of Organic Chemistry, Weizmann Institute of Science, Rehovot 7610001, Israel

**Keywords:** artificial receptors, cell surface modification, fluorescent probes, molecular cryptography

## Abstract

A method for encrypting messages using engineered bacteria and different fluorescently labeled synthetic receptors is described. We show that the binding of DNA-based artificial receptors to *E. coli* expressing His-tagged outer membrane protein C (His-OmpC) induces a Förster resonance energy transfer (FRET) between the dyes, which results in the generation of a unique fluorescence fingerprint. Because the bacteria continuously divide, the emission pattern generated by the modified bacteria dynamically changes, enabling the system to produce encryption keys that change with time. Thus, this development indicates the potential contribution of live-cell-based encryption systems to the emerging area of information protection at the molecular level.

## Introduction

**Figure 1:**
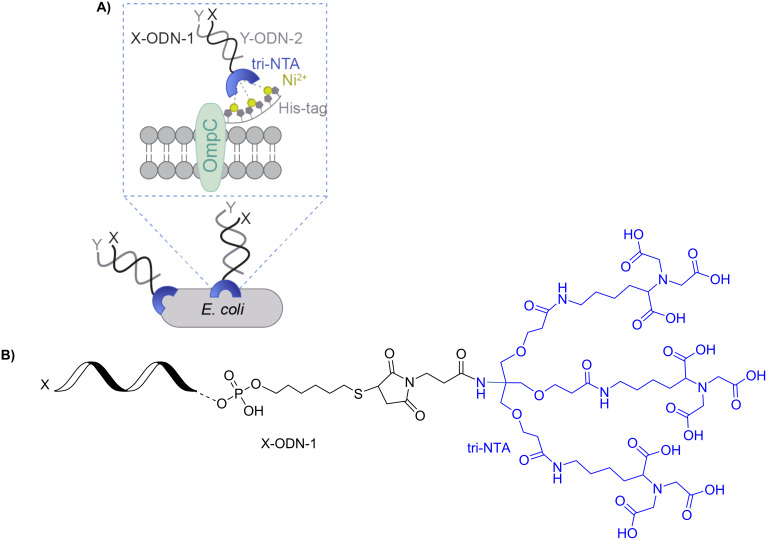
(A) Decorating *E. coli* with synthetic receptors involves the binding of X-ODN-1 to a hexa-histidine tag (His-tag) fused to OmpC. A complementary strand (Y-ODN-2) modified with the desired functionality (Y) provides the means to ‘program’ the structure of the artificial receptors. (B) Structure of X-ODN-1.

In living cells, information is processed and transferred via a series of recognition and signaling events, which normally begin by the binding of cell-surface receptors to extracellular signals, such as small molecules or proteins. In recent years, there has been considerable interest in modifying cells with artificial receptors, as a means to provide them with new properties [1]. We have recently reported a method for decorating His-tagged cell surface proteins with self-assembled synthetic receptors based on modified DNA duplexes [2] (Figure 1A). One of the oligodeoxynucleotides (ODNs) constituting the artificial receptors (ODN-1) is appended with a trinitrilotriacetic acid group (tri-NTA) that was developed by our group [3] and can selectively bind a hexa-histidine tag (His-tag). ODN-1 can also be modified with a second functional group (X), such as a fluorescent dye, to afford X-ODN-1 (Figure 1A and Figure 1B). In this way, the binding of X-ODN-1 to the bacteria will lead to the presentation of X on the cell surface (Figure 1A). A simpler way to modify the bacterial membrane is by adding to X-ODN-1 a complementary strand (Y-ODN-2) that is modified with the desired functionality (Y) (Figure 1A). In this way, the structure of the artificial receptors can be ‘programmed’ by a simple self-assembly process, which provides the means to reversibly change the properties of the cell. For example, we have shown that synthetic receptors appended with a thiol or a folate group enable bacteria expressing the His-tagged outer membrane protein C (His-OmpC) to bind to gold surfaces or cancer cells, respectively [2]. We have also shown that this approach can be used to fluorescently label the His-tagged proteins with different colors, simply by changing the dye (Y) on Y-ODN-2.

**Figure 2:**
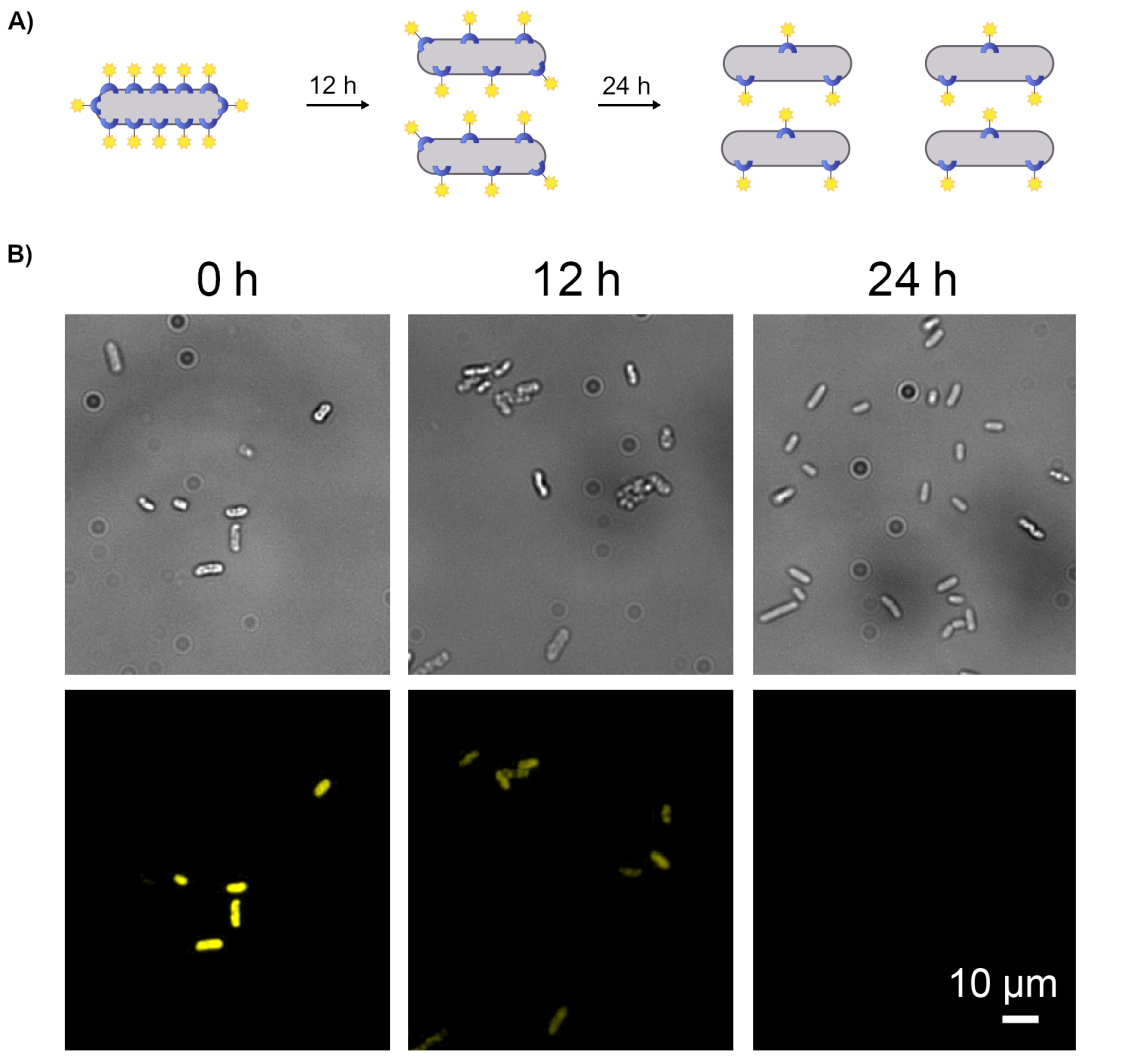
(A) Schematic illustration of the way the division of bacteria decorated with a fluorescent receptor results in a decrease in the number of synthetic receptors per cell. (B) Bright-field (top) and fluorescence images (bottom) of bacteria decorated with an ODN-1:TAMRA-ODN-2 duplex monitored at 0, 12, and 24 h.

An interesting difference between the synthetic and the natural cell surface receptors, which is in the focus of this study, is that the number of artificial receptors per cell decreases over time. This occurs because each bacterium continuously divides, which forces the synthetic receptors to split between the two daughter cells (Figure 2A). The manifestation of this phenomenon was experimentally validated [2] by observing a decrease in the fluorescence generated by the labeled bacteria during cell division (Figure 2B).

Another research direction in our group, which also involves the development of unconventional fluorescent probes, is the information protection at the molecular level [4–6]. In such studies [4–6], the emission patterns generated by the probes are used to encode or conceal data [7–8]. One potential advantage of using molecule-based security devices [7–19] over conventional electronic security systems is that the former cannot be subjected to electronic surveillance [4–6]. The small scale, versatility, and unusual operating principles are additional properties that significantly complicate finding and breaking into molecular security systems. Our main contribution to this research area, which emerged from the field of molecular logic and computing [20–23], is the creation of molecular security systems that can generate (pseudo) random fluorescence patterns [4–6]. Originally, the pattern-generating probes (or ID-probes [24]) were designed to detect multiple different analytes and their combinations [24–26]. In a later stage, we showed that the unique emission fingerprints generated by such systems can be used to secure information via different mechanisms. For example, we have used such probes to hide (steganography) [5], encrypt (cryptography) [5], and prevent access to information (password protection) [4–5]. Recently, we have constructed self-assembled, pattern-generating probes [6,27] and used them to apply the secret sharing scheme at the molecular level [6].

What significantly complicate replicating the optical codes or encryption keys that the molecular security devices produce are the various parameters that can affect the fluorescence fingerprints. For example, the emission patterns can vary as a result of changes in the type and concentrations of the chemical inputs, as well as the order by which they are introduced [4–6]. In addition, the optical signature can change as a result of alterations in the probes’ concentrations and excitation wavelengths, and the dyes that constitute them [4–6,24–26]. One parameter that was not yet applied to improve the level of defense provided by our systems is time. Unlike with some electronic security systems, in which the codes are constantly altered, or require that the user is identified within specific time frames, the optical signatures generated by our molecular security systems [4–6] remain stable over time.

**Figure 3:**
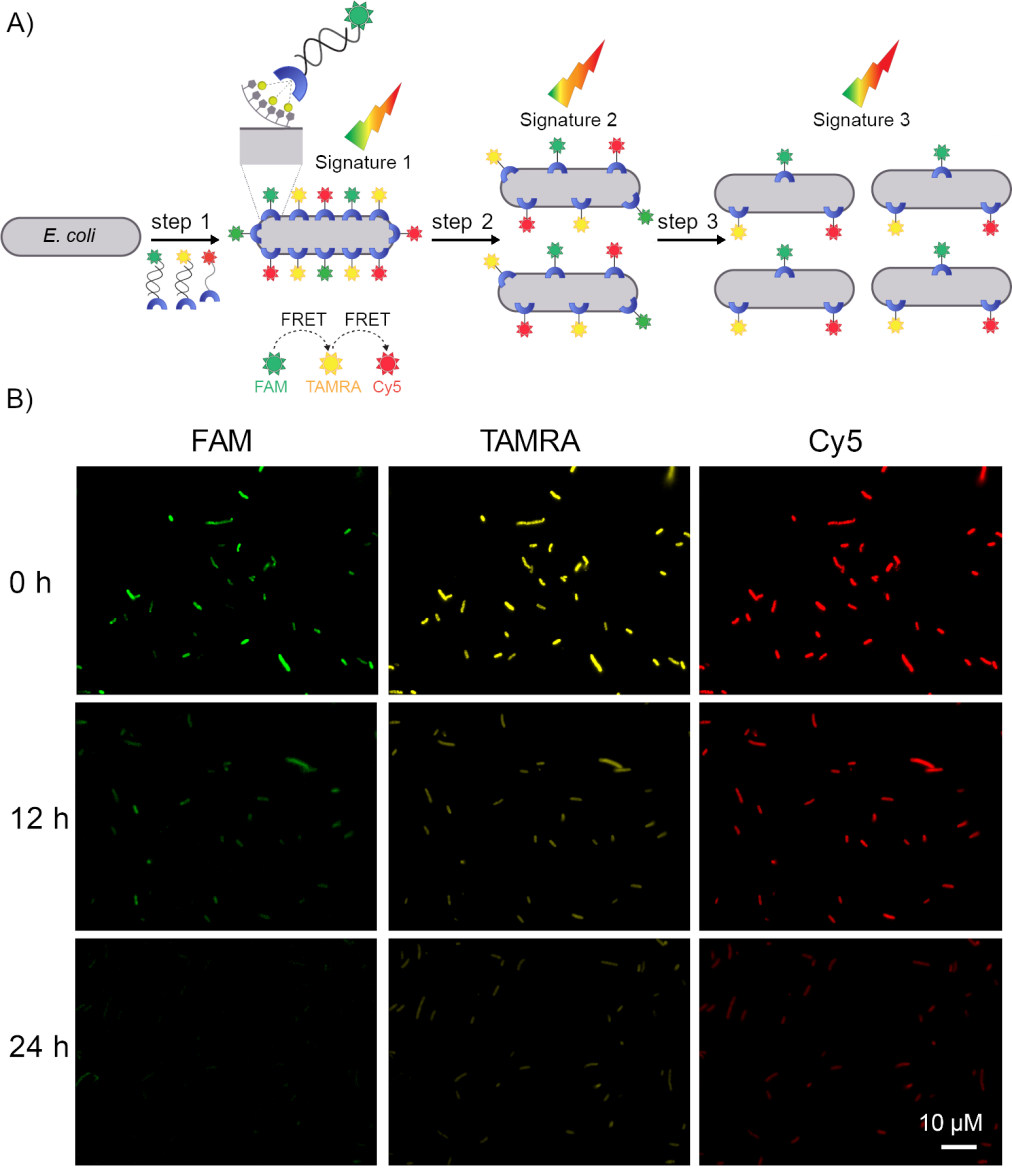
(A) Schematic illustration of the way division of bacteria decorated with three different synthetic receptors, which are appended with FAM, TAMRA, and Cy5 dyes, can change the fluorescence signature generated by the labeled bacteria. The increase in the distance between the bacteria-bound receptors decreases the FRET efficiency. (B) Fluorescence images of bacteria decorated with the DNA-based receptors monitored at 0, 12, and 24 h. In all experiments, samples that contain the same concentration of bacteria were imaged.

Realizing that the emission recorded from fluorescently labeled bacteria changes with time (Figure 2) has led us to conceive a new class of molecular security systems whose emission patterns dynamically change (Figure 3). Herein, we present the design and function of a pattern-generating system based on living cells, and demonstrate how it can be used to encrypt and decrypt secret messages in a time-dependent manner.

## Results and Discussion

### Design and operating principles

To generate encryption keys that change with time, we combined our expertise in modifying bacteria with synthetic receptors [2] and in message encryption using pattern-generating probes [5]. In our recent work, *E. coli*-expressing His-OmpC were decorated with fluorescently labeled synthetic receptors [2]. The labeling of the bacteria was obtained by incubating them with the DNA-based receptors (Figure 1) and washing off the excess of unbound receptors. The super resolution fluorescent images revealed that the bacterial membrane was densely covered [2]. Although this approach was used to label bacteria with different colors, the individual bacteria were only labeled with a single dye (Figure 2). We hypothesized that incubating the bacteria with a mixture of three artificial receptors, each of which is appended with a distinct dye, should lead to a mixed labeling of each bacterium and to the generation of unique optical signatures owing to the FRET between the dyes (Figure 3A, step 1). In addition, we expected that a division of the labeled bacteria will increase the distance between the bacteria-bound receptors, which will lead to a decrease in the FRET efficiency and to a consequent change in the fluorescence pattern (Figure 3A, steps 2 and 3).

### Fluorescence measurements

Initially, we investigated whether the bacteria can be labeled simultaneously with three different dyes. To this end, bacteria were incubated with a mixture of three DNA-based synthetic receptors (500 nM) appended with FAM, TAMRA, and Cy5 (Figure 3). The first two receptors consist of DNA duplexes assembled from ODN-1 and FAM- or TAMRA-modified ODN-2 (FAM-ODN-2 or TAMRA-ODN-2), whereas the third receptor is a Cy5-modified ODN-1 (Cy5-ODN-1) [2]. Imaging the bacteria following washing, under the excitation and emission wavelengths of the three dyes (Figure 3B), confirmed their presence on the membrane of individual bacteria, as expected from our design (Figure 3, step 1). The images also showed that, in the course of 24 hours, the fluorescence emission generated from the bacterial cells decreased (Figure 3B), as a result of bacterial proliferation (Figure 3, steps 2 and 3).

**Figure 4:**
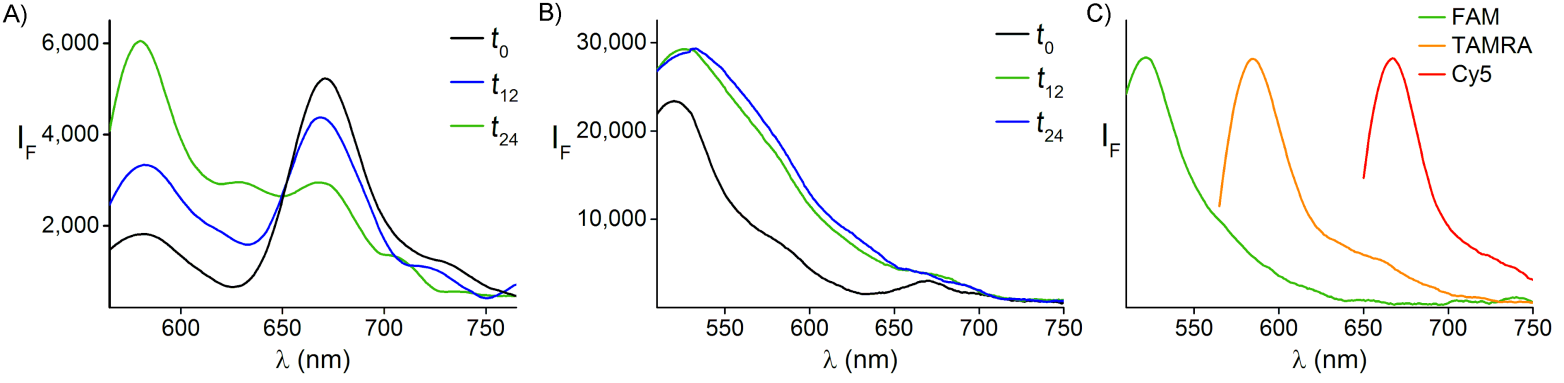
Emission spectra generated by bacteria decorated with the three different fluorescent receptors under excitation of (A) TAMRA, or (B) FAM, following 0, 12, and 24 hours of incubation. (C) Normalized emission spectra of a mixture of the three synthetic receptors in solution (in the absence of bacteria) following excitation of the FAM, TAMRA, and Cy5 dyes.

In the next step, we checked whether the binding of the receptors to the bacteria leads to the generation of emission fingerprints and, if so, whether the patterns change over time. Figure 4 shows the emission spectra generated by the bacteria-bound receptors under excitation of the FRET donors (FAM or TAMRA) following 0, 12, and 24 hours of incubation. The spectra obtained by excitation of TAMRA (Figure 4A, 545 nm) at *t* = 0 indicate the manifestation FRET between the donor (TAMRA) and acceptor (Cy5), which results in a lower emission intensity of TAMRA. Over time, with the splitting of the bacteria and the increase in the distance between the membrane-bound receptors (Figure 3A), the emission of TAMRA was enhanced and that of Cy5 decreased. Under the excitation of FAM (490 nm), the emission of the donor (FAM) also increased with time (Figure 4B). However, under these conditions, the strong fluorescence generated by the FAM-modified receptor concealed the emissions of TAMRA and Cy5; therefore, the differences between the emission fingerprints were less profound. The contribution of the bacteria to the generation of emission patterns was determined by comparing the spectra generated by the receptors in the presence (Figure 4A and 4B) and absence (Figure 4C) of bacteria. The results show that in the absence of bacteria, only the dyes that are directly excited fluoresce. This indicates that the binding of the synthetic receptors to the bacterial cells is essential for obtaining FRET between the dyes and the resulting emission fingerprints.

### Message encryption

**Figure 5:**
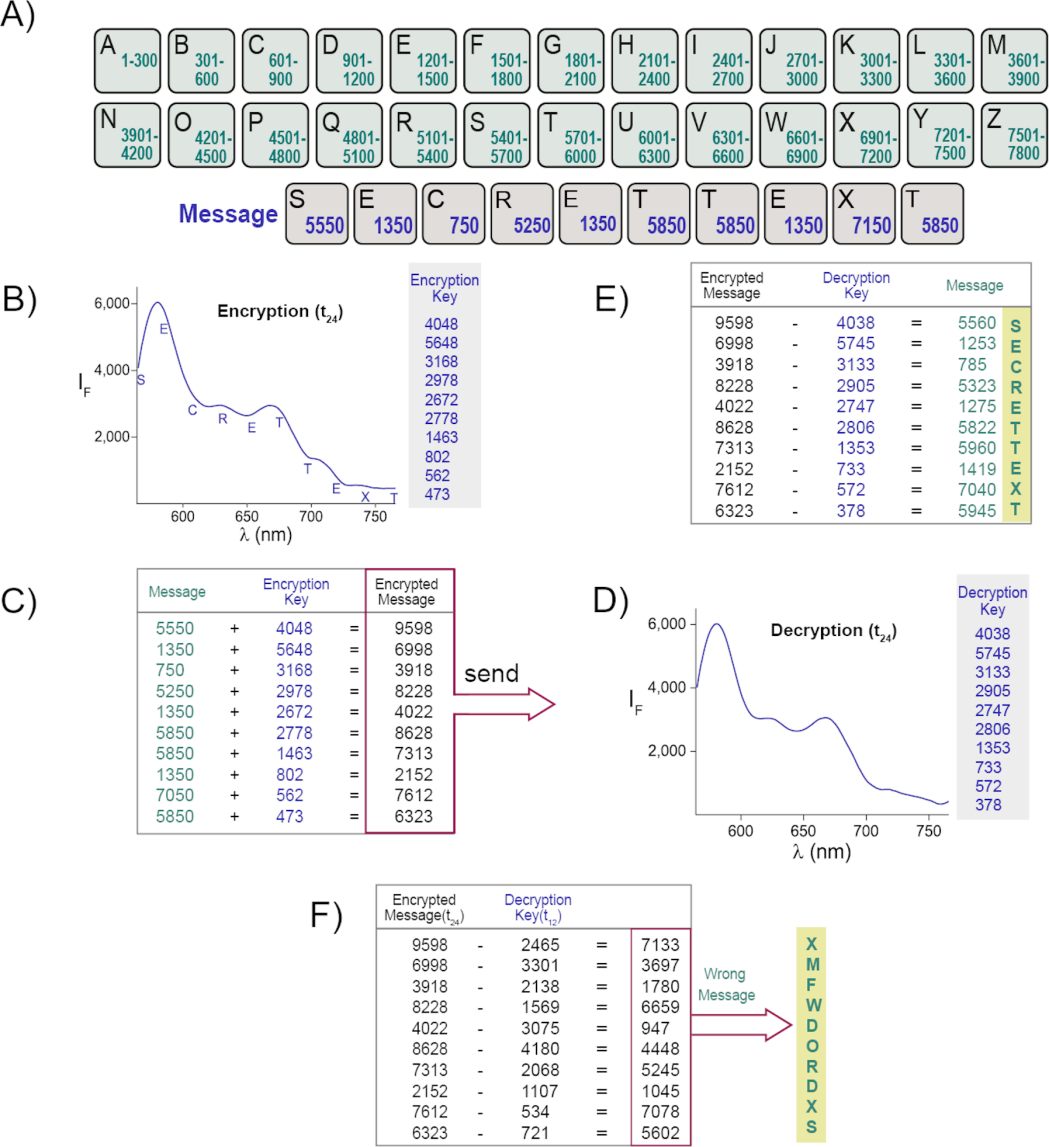
(A) An alphanumeric code. (B) An encryption key is generated by recording the fluorescence intensity values of the labeled bacteria every 22 nm. In this experiment the emission spectrum was recorded following 24 h of incubation. (C) To encrypt the message, the encryption key is added to the original message, which affords the encrypted message (cipher text). (D) To decrypt the message, the recipient needs to reproduce the emission pattern, and (E) subtract the emission intensity values from the cipher text. (F) A wrong message was obtained when using bacteria that were incubated for 12 hours after labeling.

In a recent report [5], we showed how pattern-generating fluorescent molecular probes can serve as ‘enigma-like’ cipher machines for encrypting secret messages. In the following experiment (Figure 5) we show how the use of artificial receptors and living cells as pattern-generators provides two additional layers of protection. First, whereas with the previous molecular security systems [5] reproducing the encryption keys required that the user possessed the correct combinations of molecules and chemicals, here the user must also obtain the engineered cells. Second, because the bacteria constantly grow, the emission patterns continuously change, making the encryption keys time-dependent.

To demonstrate the use of artificial bacterial receptors in encryption, we used them to encrypt and decrypt the message: secret text. Figure 5 illustrates the way the message can be converted into a cipher text (i.e., encrypted text) by the sender and then be deciphered by the receiver. This method is based on our previous study in which we used pattern-generating fluorescent probes as pseudo-random number generators [5]. Initially, the letters are converted to numbers by using a public alphanumeric code (Figure 5A). Then, an encryption key is generated by measuring the emission spectra that the labeled bacteria produce and recording the signal intensity values every 22 nm (Figure 5B). In this experiment, the emission was recorded 24 hours after bacterial labeling. To encrypt the message, the encryption key is added to the numeric message, which affords the cipher text (Figure 5C). At this point, the message is secured and can be safely sent to a recipient who possesses identical synthetic receptors and engineered bacteria, and knows the experimental conditions needed to generate the decryption key. To decrypt the message, the recipient merely needs to reproduce the emission pattern (Figure 5D) and subtract the emission intensity values (i.e., the decryption key) from the cipher text (Figure 5E). As noted before, a unique feature of the cell-based pattern-generators, when compared to our previous pattern-generating security systems, is their ability to afford time-dependent encryption keys. The way this property can improve the level of protection provided by our pattern-generating security system was demonstrated by the failure to decrypt the cipher text with bacteria that were not grown according to the sender’s instructions (Figure 5F), for example, bacteria that were grown for 12 hours after labeling (*t* = 12).

## Conclusion

To summarize, we have shown how bacteria decorated with self-assembled synthetic receptors can be used to cipher and decipher messages. Two important roles bacteria play in the encryption process have been demonstrated: First, we have shown that the FRET patterns required for encryption can be generated only in the presence of the engineered bacteria. In addition, we have shown that the bacterial growth makes the encryption key change with time. These properties significantly complicate the decryption of secret messages by unauthorized personnel.

An ultimate challenge of artificial receptors is imitating the way natural cell surface receptors process and transfer information into the cell [2]. Although it will take some time until artificial receptors will be able to engage in cell signaling pathways, this work shows an alternative way by which artificial cell surface receptors can process information. Specifically, it shows that fluorescence signals generated by such systems can be used to encrypt and decrypt messages. The use of modified living cells as pseudo-random number generators further demonstrates the potential contribution of such systems to the emerging area of information protection at the molecular level [7–8].

## Experimental

**Bacterial strains and growth conditions.** The K-12 strain KRX (Promega) was used for OmpC expression in *E. coli*. The expression of 3 copies of hexahistidine-tag at the 7th loop of the OmpC was described in our previously published paper [2]. The transformed bacteria with a His-tagged OmpC construct were cultured to saturation in LB medium supplemented with 100 μg/mL of ampicillin at 30 °C. Then, the pre-cultured cells were diluted 1:100 in fresh LB medium supplemented with the same concentration of ampicillin, and incubated until the OD_600_ reached ≈0.6. In order to induce protein expression, 0.1% rhamnose and 20 μM isopropyl-β-ᴅ-1-thiogalactopyranoside (IPTG) were added to the culture, and then the cells were allowed to grow for 12 h at 30 °C.

**Decorating bacteria with modified oligonucleotides.** The structures of the ODNs are reported in our previously published paper [2]. Samples of ODN-1:FAM-ODN-2 (duplex), ODN-1:TAMRA-ODN-2 (duplex), and CY5-ODN-1 were incubated (50 μM each) with NiCl_2_·6H_2_O (2.5 mM) in Milli-Q water for 30 min. Meanwhile, the bacterial samples were collected by centrifugation at 6000*g* for 4 min. The pellets were washed twice with M9 medium (supplemented with 2% glucose) and resuspended in 99 μL of the medium to an OD_600_ of 0.3. These bacterial cells were then incubated at room temperature for 1 h with 1 μL of a preincubated mixture of three DNA-based receptors (500 nM final concentration of each). After the incubation, the samples of the bacterial suspension were washed twice with the M9 medium and then allowed to grow in the same medium on a shaking incubator at 30 °C.

**Fluorescent imaging to study labeling during bacterial growth.** The bacterial sample to be imaged was normalized to an OD_600_ = 0.3, suspended in 100 µL PBS, and placed on a glass-bottom dish (P35G-1.5-14-C; MatTek) precoated with poly-ʟ-lysine (Sigma-Aldrich) and left to adhere for 1 h. Finally, the wells were washed vigorously with PBS three times and imaged using an Olympus IX51 fluorescent microscope. The samples were imaged using 100× objective lenses at time points of 0, 12, and 24 h.

**Fluorescence measurements and message encryption during bacterial growth.** The emission spectra of the bacterial samples labeled with the three DNA-based receptors were recorded using black flat-bottom 384-well microplates (Corning) and a BioTek synergy H4 hybrid multiwell plate reader. The fluorescence responses were measured using excitation wavelengths of 490 nm, 545 nm, and 630 nm. The experiments were performed in duplicate for bacterial samples and recorded at time points of 0, 12, and 24 h. With this procedure the encryption/decryption key was successfully reproduced four times.
